# Elective anatomy by whole body dissection course: what motivates students?

**DOI:** 10.1186/s12909-014-0272-3

**Published:** 2014-12-21

**Authors:** Annette Burgess, George Ramsey-Stewart

**Affiliations:** Sydney Medical School - Central, The University of Sydney, Sydney, New South Wales Australia; Disciplines of Surgery and Anatomy and Histology, Sydney Medical School, The University of Sydney, Sydney, New South Wales Australia

## Abstract

**Background:**

Students’ motivation provides a powerful tool to maximise learning. The reasons for motivation can be articulated in view of *self-determination theory* (SDT). This theory proposes that for students to be motivated and hence benefit educationally and professionally from courses, three key elements are needed: autonomy, competence, and relatedness. In this paper we apply SDT theory to consider medical students’ motivation to participate throughout a 2014 optional summer intensive eight week elective anatomy by whole body dissection course. The course was designed and facilitated by surgeons, and required small group, active learning.

**Methods:**

At the end of the course, data were collected from all (24/24) students by means of an open ended survey questionnaire. Framework analysis was used to code and categorise data into themes.

**Results:**

Utilising self-determination theory as a theoretical framework, students’ motivation and experiences of participation in the course were explored. Elements that facilitated students’ motivation included the enthusiasm and expertise of the surgeons, the sense of collegiality and community within the course, the challenges of group activities, and sense of achievement through frequent assessments.

**Conclusion:**

The team learning course design, and facilitation by surgeons, provided an enriched learning environment, motivating students to build on their knowledge and apply a surgical context to their learning.

## Background

Although not always considered in medical education teaching models, student motivation provides a forceful instrument to maximise learning [1]. Deci and Ryan’s (2000) interpretation of s*elf-determination theory* (SDT), proposes that for individuals to be intrinsically motivated, and hence benefit educationally and professionally from an activity, three key elements are needed: 1) autonomy, 2) competence and 3) relatedness [2]. Students need to be motivated to learn, and by trying to understand the factors that enhance students’ motivation we can better optimise learning environments and ensure future success of teaching programs. The reasons for motivation can be articulated in view of *self-determination theory* [2].

*Autonomy* refers to the students’ sense of choice and volition, whether working alone or with others [3]. Having options and choices increases students’ desire and determination to succeed [3]. Students perform best when they are intrinsically motivated to do so, rather than extrinsically motivated. In fact, offering extrinsic rewards for behaviour that is intrinsically motivated can have a negative effect on students’ motivation [4].

*Competence* refers to the students being motivated by a sense of mastery of the subject [2]. Students like to work towards mastery of a subject, and are motivated by reflective practice, as they work towards higher levels of expertise [2]. It is important that students experience an optimum level of challenge within their activities in order to remain motivated [3]. Additionally, providing students with positive feedback on tasks increases their intrinsic motivation, while negative feedback often has the opposite effect [4].

*Relatedness* refers to students’ sense of connectedness with others with similar goals and purpose [5]. This sense of connectedness is fostered when a group of students have similar ambitions and work together to attain their goals. Small group work, in particular, can promote a sense of belonging, in which individuals work closely with all of the members [6].

In this paper we apply SDT theory to consider medical students’ motivation to participate throughout an optional summer elective anatomy whole body dissection course. This is an optional course held in the final year elective component of the four year graduate entry medical program at Sydney Medical School, The University of Sydney, Australia. The course takes place on University campus, and participants are from six metropolitan clinical schools. It is an eight week, full-time, intensive, structured course, requiring substantial out of class preparation. Due to the intensive time and resource requirements of the course, only 24 places are offered annually (from a cohort of approximately 300 students), and target students interested in a surgical career. The course was designed to develop a three-dimensional appreciation of the anatomical regions of the body. The course is facilitated by senior surgeons, and requires small group, active learning. The course has been run on an annual basis since 2009, and has become so popular amongst students that applicants are required to attend a face to face selection interview with a panel of surgical dissection supervisors. The success of the course has been well demonstrated by participants’ outstanding academic results and positive feedback regarding the students’ learning expereince [7,8].

The purpose of our study was to investigate students’ motivation and experiences within the anatomy by whole body dissection course. For our study we posed the research question: “How are students intrinsically motivated during participation in the elective anatomy whole body dissection course”.

## Methods

### Context

In the 2014 program, 24 final year medical students were selected after interview to participate in an intensive 40 – day (272 hour) dissection elective over eight weeks.

### Course design

Team learning strategies were implemented [8,9]. In groups of six, students were allocated to one of four embalmed cadaver subjects. Pre-class reading from the dissection manual [10] was required for each day of dissection. Each day in class, specific group dissection tasks were clearly outlined on colour-coded spreadsheets from the manual. Each dissection group was provided with dissection instructions that had been transcribed onto laminated colour-coded cards for each anatomical region. To assist with the dissection tasks, a central console was used to provide projected images from the dissection manual. The Supervisors held frequent SCORPIOs, designed to provide students with a problem-based, integrated learning experience, and frequent formative and summative in-class tests and practical assessments. Formative assessments were held daily. Formal, standardised practical assessments, requiring identification of 20 anatomical structures on wet specimens were carried out pre-course, mid-course, end-course and post-course. A total body dissection was completed by all students.

The daily program consisted of a daily dissection briefing given by an allocated student utilising diagrams; allocated dissection tasks on cadavers in the wet lab reinforced by small group wet prosected specimen demonstrations; a 30 minute lecture by a supervisor on the clinical applications of the anatomy being dissected; and completion of the allocated dissection tasks for that day.

### Supervision

The course was facilitated by nine highly experienced senior surgeons from various specialities, known as “Supervisors”. The Supervisors were present for their area of anatomical expertise. Two to three supervisors were present at any one time during dissection. Nine surgical trainees were also recruited as Demonstrators.

### Data collection

Data were collected by survey questionnaire from students at the end of the course. The questionnaires were distributed by the first author, who has no involvement in teaching or administration of the course. All 24 students were asked to complete a questionnaire regarding their experience in the course. The questionnaire included open ended questions aimed at eliciting responses from students regarding their motivation in relation to the course design, teaching methods and supervision.

### Data analysis

Framework Analysis was used to code and categorise data into themes [11,12]. It was noted that emergent themes in the dataset resonated closely with key constructs within self-determination theory [2]. Subsequently, SDT theory was used as a conceptual framework to identify recurrent themes. A coding framework was developed to code the entire dataset through the theoretical lens of SDT theory.

Ethics approval was obtained from the University of Sydney Human Research Ethics Committee. Written consent was gained from each study participant.

## Results

### Student demographics

A total of 24 final year medical students (18 male and 6 female) with a mean age of 27.4, SD 3.07, range 24 to 32 years, participated in the course. All students held a prior degree, including degrees in Pharmacy, Engineering, Law, Science, Dentistry and Business.

### Survey responses

All 24 students (100%) completed the questionnaire at the end of the 2014 course.

Utilizing *self-determination theory* as a theoretical framework, we illustrate the student’ motivation and experiences of participation in the anatomy by whole body dissection course. Table 1 displays student motivation in relation to autonomy; Table 2 in relation to competence; Table 3 in relation to relatedness.

**Table 1:** **Student motivation in relation to** ***autonomy***

**THEMES RELATING TO AUTONOMY**	**STUDENT COMMENTS**
**Students were undertaking the course of their own volition, and applied themselves academically because they wanted to, not because it would go on their transcript.**	*“The fact that the course is voluntary and that the marks have no impact on my degree enables me to approach the course with a proud sense of achieving above and beyond the basic demands of the course”.*
	*“The pressure to perform for marks is only out of desire to learn and not for a number on your CV”.*
**Self directed learning tasks promoted individual and group autonomy.**	*“There is a set reading for each night from Cunningham’s Dissection manual, but the course provides you with the freedom to learn from whatever resources you find useful. The wide range of external resources broadened my appreciation of surgical anatomy and my understanding of medicine as a whole”.*
	*“The combination of set reading for each evening plus a chance to apply and reinforce the knowledge the following day gives a very strong incentive to keep up with the reading and strongly reward independent study. There were different learning strategies employed across the group to cover the material. (i.e. we were not told how to learn it).”*
**Students felt that they high quality of teaching, and the particular balance between structured teaching and self-directed learning.**	*“Because the quality of teaching in this course is high, self-directed learning tasks are beneficial”.*
	*“We were able to do the activities, but would ask for help if we needed it”.*
**Team members collaborated with each other, and with their supervisors to shape the quality of their learning.**	*“Everyone put in effort in the group and played their roles well (i.e. someone looking up books/pictures) whilst the others dissected. The supervisors made it fun and lighthearted which bred a sense of collegiality”.*
	*“Having mini tutorials in groups of six makes the learning more individualized. Independent learning is fostered with each tutor providing us with individualized feedback throughout the course”.*
**Students felt they were treated as equals by supervisors, given clear outcomes, but the freedom to explore areas throughout the day that they needed to.**	*“There is a genuine sense of partnership from the senior tutors, assuming we will commit to the effort required and so treating us as adults embarking on a journey, rather than students who need to be controlled to be taught”.*
	*“We were not constantly supervised, we were able to break for self-directed learning during the day, enabling us to answer any burning questions and explore unknown or taxing areas”.*

**Table 2:** **Student motivation in relation to** ***competence***

**THEMES RELATING TO COMPETENCE**	**STUDENT COMMENTS**
**The interactions with supervisors that provided a clinical context, and the 3D structures helped to develop student competence.**	*“The supervision and teaching methods help to correct any misconception you may have after studying the textbook”.*
	*Fantastic teaching with fantastic clinical insights. The teaching methods of questioning relevant material and a Socratic approach to clinical reasoning was hugely beneficial”.*
	*“ Reading the material in the books helps you become familiar with the terminology but seeing it all in 3D in the cadaver helped me really understand the anatomy and its relation to each other”.*
**Supervisors were careful to match their level of teaching to the level of student ability. At the same time, however, supervisors had high expectations of students.**	*“Teachers have the ability to be flexible and adapt your methods to the strengths of your team”.*
	*“All of the supervisors, demonstrators and past students were very positive in their approach, but also expected a lot. This makes the course a big challenge, but an achievable and very positive one and brings the group of students and teachers closer together”.*
**Students found a sense of accomplishment in helping each other.**	*“The group learning was valuable as all members in the team had different levels of knowledge to begin with and different areas of key interest. As a result we were able to assist each other and provide clinical detail to the anatomy as well. I think this was one of the strongest components that helped to facilitate my learning”.*
	*“There is a real atmosphere of teamwork and team achievement, especially regarding exam results with students cheering each other's success”.*
**Frequent and early assessment, and immediate feedback allowed students to identify their own weaknesses early, benchmark against each other, allow reflection, and target areas for improvement.**	*“At a very early stage my weaknesses were made very clear through assessment. Reflection and motivation at this early stage gave me a realistic opportunity to address them and improve (before it was too late). Each assessment gave me a clear unbiased appreciation of my progress and directed my future learning for the next element”.*
	*“Frequent assessment is AMAZING. You are constantly refreshing and testing your knowledge. Marks are made public, so there is a sense of competition and you constantly try to get the top mark. Public marks motivate me to study harder. The tutors are frank when you don't know things you should but give praise when its warranted”.*
**The constant repetition of questions, lectures, exams and SCORPIOS helped students to remember.**	*“Repeated questioning. Repeated examinations. Repeated SCORPIOs. ‘Repetition is the mother of learning’ “*
	*“Constant repetition during tutes, lectures, and SCORPIOs really helped cement things in my brain. Having tutors roam around different tables, giving mini-tutes and asking questions ensured that by the end of the day I usually felt I had learnt a substantial amount of anatomy and more importantly that I would not forget it easily”.*
**The daily SCORPIOs consolidated the students’ knowledge, identify gaps in learning, and directed learning towards what was important to learn.**	*“The SCORPIOs at the end of the day were an excellent way to demonstrate and reinforce knowledge gained, plus they highlight the key surgical take home points in a competitive yet non-stressful environment….directed my attention to what was most important to understand”.*
	*“SCORPIO at the end of the days helped to consolidate learning and showed me what I understood and where the gaps are. Being asked questions repeatedly is a great way to find out what you do or do not know”.*

**Table 3:** **Student motivation in relation to** ***relatedness***

**THEMES RELATING TO RELATEDNESS**	**STUDENT COMMENTS**
**Students felt that everyone, including students, demonstrators and supervisors, were all there for the same purpose – to help each other to learn.**	*“You develop a friendship with your group and supervisors as we were all there with the same intentions, to help each other learn anatomy. There was always a feeling of friendliness and that you can approach anyone for help to learn”.*
	*“We were all working together. We have a single aim and all helped each other achieve that aim. The regular testing gave us a clear set of goals. Furthermore, we wanted to make the Supervisors proud with decent results. He gave us praise for mastery of anatomy and that encouraged us to master the next topic”.*
**Students felt that they were part of a professional (surgical) community.**	*“In addition, the senior tutors engaged with us on a very equal level, promoting a sense of inclusion within the surgical society”.*
	*“The past students returning as tutors really showed how it’s kind of like a big family, with their advice about how to pass primaries and get onto training programs being invaluable”.*
**Shared practices, teaching methods, such as scorpios and frequent quizzes helped to create a shared learning experience for students.**	*“The SCORPIOs, although they single people out, enhance collegiality because you don't like to see a group member fail. The placement of the white boards near the groups also seems to really encourage group learning and leading by those in the group who know more than others”.*
	*“The overall spirit that is instilled in everyone really is highlighted by constant quizzing of each other in addition to that by the demonstrators”.*
**The enthusiasm and dedication of the supervisors cultivated a rich learning environment.**	*“All students are motivated and we all want to learn and be the best. This fosters an honest and productive learning environment. All teachers and demonstrators have an amazing ATTITUDE - they want and demand that we learn. Their dedication and time and high expectations of us push us to learn, drive us to do the extensive readings each night and be the best we can. Seeing 80 year old surgeons come back and teach us is amazing. This circular learning process is what medicine is about and further adds to our feeling of collegiality”.*
**The difficulty of the challenge and the need to problem solve brought students, demonstrators and supervisors together.**	*“The course is demanding, so there is a shared bond through adversity. The sense that we are all in it together (including the demonstrators) is palpable”.*
	*“The content is tough and there is so much to learn that this does create a shared experience and a sense of belonging. More proficient students are keen to help those who are less so and the supervisors are keen to assist at all times in dissection and activities. The assessments provide a competitive drive to perform well”.*
**Working in small groups prompted collegiality. Heterogenic formed groups meant that students had different levels of understanding and helped each other.**	*“We worked together. Having one cadaver meant that we all needed to be on top of our game. If one member was deficient in an area the group was motivated to work together to ensure we all understood and you were able to explain the information”.*
	*“I have made some life-long friends and colleagues during this course. The satisfaction of learning something new or teaching something with a colleague develops stronger bonds and also helps both parties achieve a joint goal”.*

## Discussion

According to SDT theory, the three key elements in optimising one’s motivation are autonomy, competence and relatedness [2]. These elements engage one’s motivation from within, producing desirable benefits for learning and behaviour. Each of the elements is considered below in the context of students’ feedback regarding their participation in the anatomy whole body dissection course:

### Autonomy

According to the SDT theory, it is more motivating for students if they feel they are acting through their own volition [2]. Certainly, participating students freely chose to undertake the elective dissection course. Rather than choosing an overseas elective placement, individual students came together from six different clinical schools with the joint purpose of developing surgical anatomical skills. Although their grades would not appear on their transcripts, they were driven to achieve high marks for themselves, their teams and their supervisors. Students commented on a “*desire to learn and not for a number on your CV”. Autonomy* doesn’t mean working separately from others, but rather, using information to guide decision making [13]. The course design provided students with self-directed learning activities, so that students worked in collaboration with their team members, other teams, and supervisors, to make the dissection activities meaningful and worthwhile. With guidance to required information, the curriculum was open for students to shape the quality of their own learning [14]. Autonomy was fostered within the dissection room, where students had opportunities to engage with others and make choices within clear and structured guidelines [13,15].

### Competence

Students like to feel that they are *masters* of what they do, and a sense of competence was cultivated when they attained particular knowledge and skills [2]. As one student commented, *“There is a real atmosphere of teamwork and team achievement, especially regarding exam results with students cheering each other’s success”.* Importantly, complex interaction between the supervisor and the students developed student autonomy and competence [2]. It is important to match levels of competence with levels of supervision [16]. By allowing students to undertake the dissections, with appropriate supervision, their responsibilities were aligned with their abilities, and a sense of competence was developed [9]. One student commented that supervisors had *“the ability to be flexible and adapt… to the strength of your team”.*

Students need to be able to be successful at the tasks at hand. However, in order to remain motivated, they need to have higher levels of proficiency to which to aspire [2,17]. Students in the anatomy dissection course felt a sense of accomplishment when they finished the assigned tasks for the day, and progressively improved on class tests. Throughout the dissection course, there was no shortage of frequent and affirmative feedback from supervisors. Informal formative assessments were held frequently, acting as incentives for “the pursuit of excellence”, with students receiving regular feedback. Well-defined goals were set. By holding formal standardised practical assessments at four time points across the eight week course, students were able to see their progressive mastery of the course, and could identify specific areas in need of improvement. As students continued to develop their dissection skills along with a sense of mastery, motivation was fuelled, with reflective practices leading to continual improvement in the task [2,13]. As well as the wet specimen identification assessments, students completed MCQ and Spot Test assessments at the end of each regional assessment. Doing well in the tests reinforced a sense of acquiring competence.

### Relatedness

Relatedness refers to a sense of joint *purpose* to the task. SDT suggests that people need to feel connected, and this may be fostered within groups with the same ideals and goals [5]. Throughout the dissection course, students were made to feel part of a professional community [13]. As one student commented, *“You develop a friendship with your group and supervisors as we were all there with the same intentions, to help each other learn anatomy.*” Surgical supervisors contributed their stories and examples to provide relevant surgical clinical contexts, contributing to students’ understanding of topographical human anatomy. In these social surroundings, students were able to construct their own detailed knowledge. They developed their own shared practices, such as impromptu quizzes with senior staff. The “*overall spirit*” of the supervisors and the participants shaped the culture of the learning environment, and helped create a relaxed, collegial atmosphere [13]. Central to SDT theory, a sense of belonging [18], was fostered within student teams and the class itself. Students worked on problem solving group activities, alongside each other, tackling challenging tasks and solving problems together. Students had a joint sense of purpose to master the course [5,19].

### Significance of the study

Use of an existing theoretical framework may assist medical education researchers in understanding and determining the value of the presented research. According to McMillan [20], an educational research study should display consistency with established theory, building on what is already known [20]. By utilizing self-determination theory as a theoretical lens to interpret and understand the data, we have strengthened our study. SDT theory may provide a useful framework to demonstrate students’ motivation to take part in other medical education courses.

### Limitations of the study

One limitations of our study is that it is a small scale study. Although 24/24 (100%) of students completed the surveys, this is a small number. Also, it should be acknowledge that the students attending the anatomy by whole body dissection course had voluntarily chosen to do so, and therefore were more likely to put effort into this endeavor, and perform well academically.

## Conclusion

The course design and supervision of the anatomy dissection course provided an enriched learning environment that motivated students to build on their knowledge together and apply a surgical context to what had been learnt. The three key features of SDT – autonomy, competence and relatedness, drove students’ desire to achieve. Students undertook the intensive course of their own volition, and were highly motivated by the enthusiasm and expertise of the surgeons; the sense of collegiality and community within the course; and the continuous challenges of group problem solving activities, and the sense of achievement through frequent assessments. By developing a better understanding of the influences on students’ motivation, future course designs may be improved to optimise student learning.
